# Automatic resin duct detection and measurement from wood core images using convolutional neural networks

**DOI:** 10.1038/s41598-023-34304-7

**Published:** 2023-05-02

**Authors:** Anna Fabijańska, Gabriel D. Cahalan

**Affiliations:** 1 Institute of Applied Computer Science, Lodz University of Technology, 18 Stefanowskiego Str., 90-537 Lodz, Poland; 2 The Nature Conservancy, 425 Barlow Place Suite 100, Bethesda, MD 20814 USA

**Keywords:** Environmental sciences, Engineering

## Abstract

The structure and features of resin ducts provide valuable information about environmental conditions accompanying the growth of trees in the genus *Pinus*. Therefore analysis of resin duct characteristics has been an increasingly common measurement in dendrochronology. However, the measurement is tedious and time-consuming since it requires thousands of ducts to be manually marked in an image of an enlarged wood surface. Although tools exist to automate some stages of this process, no tool exists to automatically recognize and analyze the resin ducts and standardize them with the tree rings they belong to. This study proposes a new fully automatic pipeline that quantifies the properties of resin ducts in terms of the tree ring area to which they belong. A convolutional neural network underlays the pipeline to detect resin ducts and tree-ring boundaries. Also, a region merging procedure is used to identify connected components corresponding to successive rings. Corresponding ducts and rings are next related to each other. The pipeline was tested on 74 wood images representing five *Pinus* species. Over 8000 tree-ring boundaries and almost 25,000 resin ducts were analyzed. The proposed method detects resin ducts with a sensitivity of 0.85 and precision of 0.76. The corresponding scores for tree-ring boundary detection are 0.92 and 0.99, respectively.

## Introduction

Resin ducts or canals are a feature of trees in the genus *Pinus *(and other genus including *Larix, Picea, Psuedotsuga, and Shorea*). Their structure and properties provide valuable information about environmental conditions in the corresponding years of tree growth. E.g., resin ducts have been shown to correlate with mortality from beetles and drought in some *Pinus* species in the USA^1,2^. Also, local climatic adaptations affect duct characteristics and the ability to deliver chemical and physical defenses through oleoresin^3^.

Although analysis of the resin ducts has been an increasingly common measurement in dendrochronology, this potential source of valuable information is difficult to obtain. The reason is that no tool automatically recognizes the resin canals and analyzes their properties in relation to the tree rings to which they belong. Thousands of ducts must be manually marked in an image of an enlarged wood surface to obtain reliable measurements. Time series of resin duct measurements are tedious and time-consuming to record using manual methods^4^. The measurement results may vary depending on the skills and perception of the operator.

To our best knowledge, no method exists that would automatically detect, measure and output a time series of resin ducts in wood images. Nevertheless, attempts to automate some parts of the detection and analysis of resin ducts have already been proposed. These are mainly semi-automated tools in macros coupled with ImageJ (or other software) that partially automate the analysis of resin duct properties. These approaches either require manual pre-selection of resin ducts or use simple image processing methods to detect the resin ducts. However, traditional image processing methods are often inadequate for reliably detecting candidate resin ducts and tree ring boundaries. The variability in the appearance of these structures, which can depend on factors such as tree species or environmental conditions, makes defining precise rules for feature detection using thresholding challenging. Furthermore, interference from other wood image features, such as bark, knots, or wood fibers, can further complicate feature detection using classical image processing algorithms, resulting in low detection accuracy, missed detections or false positives.

The earliest semi-automatic method for identifying resin canals and quantifying their properties through tree-ring-based statistical measures was proposed by Chen et al.^5^. The approach uses color-scanned images of the transverse surface of polished wood samples and incorporates color constraints and shape-based filtering for resin canal selection. The reported resin canal detection rate is low (68%), still requiring extensive visual inspection and manual correction of false or missing resin canals.

The approach proposed by Thomas and Collings^6^, which counts resin channels using images acquired with a commercial film scanner modified to operate with circularly polarised light, has similar drawbacks. Their image analysis procedure uses basic image processing (i.e., intensity thresholding followed by morphological processing) to detect candidate resin canals. Particles are then counted using experimentally selected thresholds for size and circularity to obtain basic statistics on resin canals.

The most recent study described by Hood et al.^7^ quantifies user-detected resin ducts via an R script that computes three unstandardized and two standardized resin duct metrics. The ducts must be manually approximated by an ellipse of size and shape that an operator in the ImageJ software adjusts. In addition, the authors recommend using CooRecorder to measure ring widths, CDendro to generate ring width chronologies, and COFECHA to check the accuracy of cross-dating. Although such a procedure makes it possible to relate the statistics of the resin ducts to the corresponding tree rings, it is cumbersome because it requires switching between several different types of software and the representation of measurement data. Standardization of terms and metrics via the tree ring properties has been suggested to further improve the addition of resin ducts^4,8^.

In parallel, the possibility of automating other tasks related to wood technology and wood image analysis has been explored over the past decade. Specifically, deep learning (DL) and convolutional neural networks (CNN) have revolutionized wood technology in recent years by providing exceptional capabilities for processing large amounts of data, identifying patterns, and making predictions^9,10^. The CNN models have mainly been trained to identify wood species automatically^11^. With a motivation to preserve endangered species, prevent illegal logging, and ensure the authenticity of wood products, DL solutions have been proposed to recognize wood species from images of wood surface^11–17^, standing trees species recognition from 3D point clouds of trees collected by light detection and ranging (LiDAR) or terrestrial laser scanning (TLS)^18–20^ and near-infrared (NIR) spectroscopy based tree species identification^21,22^. Convolutional neural networks have also been proposed to process remote sensing and aerial images in order to monitor forest infestation and health conditions or detect fires^23–28^. Deep learning has also entered automatic wood quality control and nondesctructive testing technology, where convolutional neural networks have been proposed to detect defects and anomalies in wood products, such as wood knots, dead knots, cracks, splits, or pest damages^29–37^, wood composites failure predication^35^, wood log tracing^38^ or categorizing the damaged wooden elements^39^.

Despite these advancements, the use of machine learning and CNNs in dendrological analyses is limited. Existing works have focused on the automation of pitch detection^40,41^ and delineating tree ring boundaries and measurements to improve the workflow in dendrochronological studies^42–49^. Therefore, this paper aims to explore the performance of CNNs in the resin duct detection task. Specifically, we propose and test a method that uses a CNN model to automate the detection of resin ducts in scans of tree ring samples and output measurements and metrics as a time series. Using a CNN for resin duct detection offers several advantages over traditional image processing methods, including learning complex features automatically, simultaneous detection of multiple features, and improved performance in challenging imaging conditions or when analyzing wood samples with diverse properties.

The contributions of this paper are as follows:

- The first fully automatic method for detecting resin duct in wood core images is proposed. The method is underpinned by a convolutional neural network applied in a patch-based setup.
- A tree ring segmentation approach is proposed, which comprises a convolutional neural network for tree ring boundary detection, watershed transform, and region merging to identify connected components corresponding to successive rings.
- The above two methods are integrated into an image analysis pipeline that quantifies the properties of resin channels in terms of the tree ring to which they belong. This extends previous automated methods that only considered ring widths and allows standardization of resin duct measurements as proposed in Hood et al.^4^ and Vázquez-González et al.^8^

## Materials

**Figure 1:**
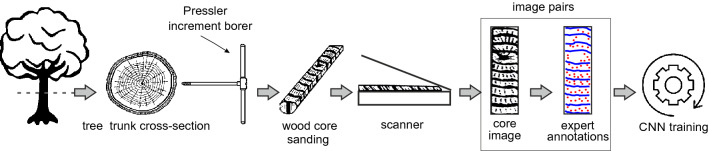
Data acquisition and preparation workflow for training the convolutional neural network.

In this study, the wood core images and the corresponding image data were acquired through the procedure summarized in Fig. 1.

Five *Pinus* species were considered, i.e., *Pinus palustris* Mill. (PIPA2 or hereafter PIPA), *Pinus virginiana* Mill. (PIVI2 or hereafter PIVI), *Pinus rigida* Mill. (PIRI), *Pinus taeda* L. (PITA), *Pinus pungens* Lamb. (PIPU5 or hereafter PIPU). In total, 74 cores were collected. To obtain these samples, 54 trees were cored with a 5.15 mm increment borer at locations across the state of Maryland and one site in the state of Virginia in the USA. Multiple cores were obtained from the same tree in some cases, while at other times, single cores were obtained. Each core in this study was treated as a single separate sample because the wood features of interest are unique in each core. Particularly, wood samples were selected to vary in density and width of tree rings, appearance of resin ducts, and wood texture. Cores were prepared following a standard procedure^50^. Specifically, core samples were mounted and prepared using successively finer sandpaper from 120 to 800 grit using a belt sander to obtain a flat surface. The sanding process increased discernibility of tree ring boundaries and resin ducts. Cores were then scanned using a flatbed scanner at 2400 dots per inch. The spatial resolution of the images ranged from 244 $\times$ 6990 to 410 $\times$ 27666 pixels.

The expert in dendrochronology manually annotated the tree-ring boundaries and the resin ducts in each wood core image. A combination of a touchscreen-style monitor with a stylus and mouse was used to draw circles and lines over the resin ducts and tree ring boundaries on the scan files. This procedure resulted in 8024 tree-ring boundaries and 24,735 resin ducts identified and annotated by an expert. Their distribution among the considered *Pinus* species is summarised in Table 1. Also, the time spent by an expert when annotating the data was recorded (cf “Results” Section).

**Table 1:** Summary of a dataset of wood core images.

Species	Num. cores	Num. rings	Num. resin ducts
PIVI	18	1769	4727
PITA	13	1261	4850
PIRI	30	3696	10756
PIPU	10	1247	3632
PIPA	3	114	770
total	74	8024	24735

The dataset of expert-annotated images was subsequently divided into three folds, with each considered species being more or less equally represented. These folds were employed for the three-fold cross-validation of the proposed pipeline. Specifically, two folds were utilized for training a convolutional neural network (CNN), and one fold was reserved for testing. This process was repeated three times, with different folds for training and testing in each repetition.

## The proposed approach

### General overview

**Figure 2:**
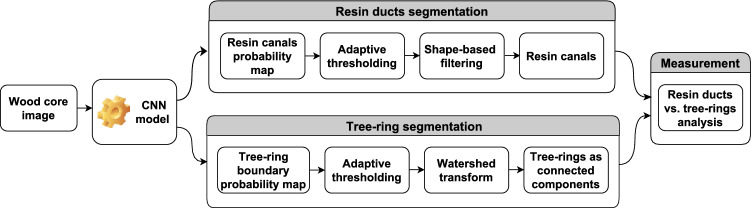
Pipeline of the proposed approach.

The proposed method aims to automatically obtain measurements of the resin canals versus tree rings from images of the wood samples. It transforms an input wood core image $\mathscr {I}(x,y)$ into two labeled images $\mathscr {R}(x,y)$ and $\mathscr {D}(x,y)$, such that $\mathscr {R}(x,y): \Omega \subset \mathbb {R}^2 \rightarrow \{1, 2, \ldots , K\}$ is composed of *K* connected components $r_i = \{(x,y): \mathscr {R}(x, y) = i\}$ each corresponding to a tree ring where $\mathscr {R} = \big [\bigcup _{i=1}^K r_i\big ]$ and ${r}_i \cap {r}_j = \emptyset$ for $i \ne j$, and $\mathscr {D}(x,y): \Omega \subset \mathbb {R}^2 \rightarrow \{0, L\}$ is composed of $L+1$ connected components $d_i = \{(x,y): \mathscr {D}(x, y) = i\}$ corresponding to resin ducts ($i>0$) and background ($i=0$), such that $\mathscr {D} = \big [\bigcup _{i=0}^L d_i\big ]$ and ${d}_i \cap {d}_j = \emptyset$ for $i \ne j$. For this purpose, a pipeline sketched in Fig.  2 is proposed.

The proposed approach builds on the convolutional neural network (CNN) to detect resin ducts and tree-ring boundaries. The Attention UNet (AttUNet) model is incorporated into the pipeline to generate joint probability maps of the abovementioned structures. The maps are next subjected to postprocessing, which aims to define the precise location of resin ducts and segment consecutive tree rings. Finally, the statistics of the resin ducts versus the corresponding tree rings are determined. A detailed description of the consecutive steps of the pipeline is provided in the following subsections.

### CNN model

#### Architecture

The CNN model used in this paper extends the author’s earlier study on tree-ring boundary detection using the U-Net model^48^ and derives from the Attention U-Net originally proposed in^51^. Specifically, the model needed to be adapted to the unconventional image resolution of the core images considered in this study. Since core images vary in size and their length is significantly bigger than the height, the model was applied in a patch-based setup to consecutive image tiles. Also, the model was extended by incorporating attention mechanisms to enhance further the model’s ability to focus on important image features related to resin ducts and tree-ring boundaries while ignoring irrelevant ones.

The final model used in this study represents encoder-decoder architecture $\mathscr {N}: \mathscr {I}(x,y) \rightarrow \mathscr {P}(x,y)$ which transforms input wood core images $\mathscr {I}$ into probability maps $\mathscr {P} = [\mathscr {P}_R, \mathscr {P}_D, \mathscr {P}_B]$ of structures the model aims to detect such that $\mathscr {P}_R(x, y)\in [0, 1]$, $\mathscr {P}_D(x,y)\in [0,1]$, $\mathscr {P}_B(x,y)\in [0,1]$, and $\mathscr {P}_R+\mathscr {P}_D+\mathscr {P}_B = \textbf{1}$. The model was trained to detect two types of objects, i.e. to predict probability maps of resin ducts $\mathscr {P}_D$ and tree-ring boundaries $\mathscr {P}_R$. Additionally, a background probability map $\mathscr {P}_B$ is generated as a side effect.

**Figure 3:**
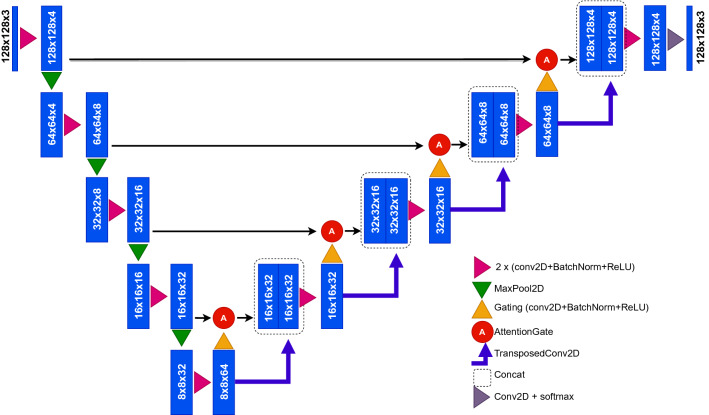
A modified Attention U-Net model used in this study.

The model’s input size was set to 128 $\times$ 128 pixels, which is the largest power-of-two-based size that could be obtained from all the core images used in the study. However, since this input size is smaller than the input size used in the original AttUNet model, the number of filters in each filter block was reduced to downscale the model. To determine the optimal downscale level, the original number of filters was gradually reduced by a factor of two while monitoring the model’s accuracy. Reducing the number of filters was stopped when the model’s accuracy visibly decreased compared to the original setting. This resulted in a downscale factor of four.

The resulting model comprises two paths (see Fig. 3). The input image is gradually downsampled in the contracting path by max-pooling layers that follow repeated applications of two convolutional blocks. The latter comprises the convolutional layer, followed by batch normalization and ReLU activation. The number of filters in the convolutional block doubles with each pooling. It starts with four filters in the first convolutional block, while in the deepest convolutional layer of the contracting path, the number of filters equals 64. Compared to the original AttUNet model, the number of filters in each convolutional layer was thus reduced by a factor of four.

The expanding path is symmetrical, with pooling layers replaced by the image upsampling by a factor of two, accompanied by halving the number of filters in each layer. The last layer incorporates softmax activation that outputs the probability of each pixel belonging to a resin duct or tree-ring boundary for each pixel.

The feature maps from the expanding path are concatenated with their counterparts from the contracting path via the skip connections to facilitate image upsampling. The latter ones incorporate soft attention gates, which focus more on the image areas of high relevance and suppress activations in irrelevant regions.

#### Training

**Figure 4:**
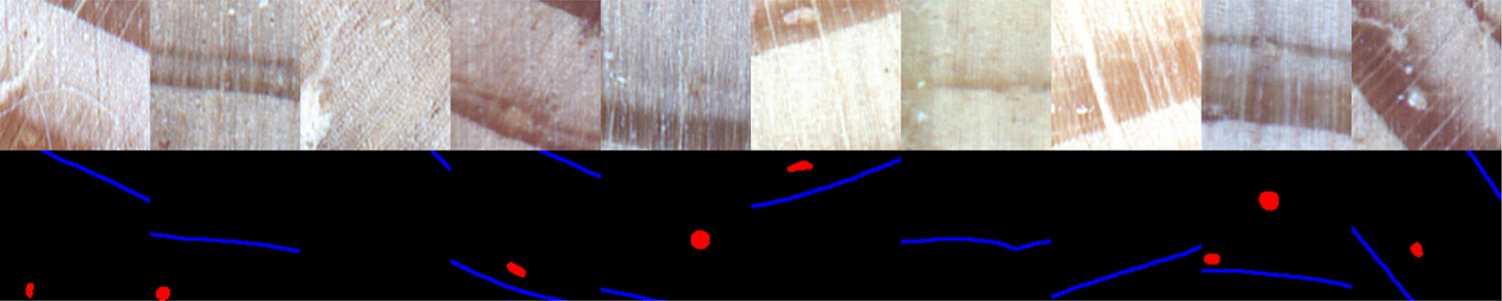
Sample train patches; upper panel—original wood images; bottom panel—ground truths for tree-ring boundaries (blue) and resin ducts (red).

The model was trained with pairs $(\mathscr {I}, \mathscr {G})$ of image patches representing wood core $\mathscr {I}$ (input to the model) and the corresponding resin ducts and tree-ring boundaries marked with different colors $\mathscr {G}$ (output of the model). Since the images of wood core images considered in this study are of varying and not typical spatial resolution (with image length tenths times greater than its height), the CNN model was applied in a patch-based set-up. For training the model, 1500 patches $p_i = (p_{I_i}^{(x,y)}, p_{G_i}^{(x,y)})$ of size 128 $\times$ 128 pixels were sampled randomly from each train wood core image ($p_{I_i}^{(x,y)} \subset \mathscr {I}$) and expert annotated image ($p_{G_i}^{(x,y)} \subset \mathscr {G}$) from the corresponding locations (*x*, *y*) resulting in about 74000 train patches in total. The patch size was selected experimentally. It was ensured that each train patch contained at least one of the structures of interest. Sample pairs of training patches are presented in Fig. 4.

The train patches were randomly divided into a train (80%) and a validation set (20%). Since sampling of the train patches was quite dense, artificial data augmentation was used to avoid model overfitting. The transformations included vertical and horizontal flipping, rotation, and shear of a small range.

The model was trained on GeForce GTX TITAN X GPU equipped with 12 GB of DDR5 RAM and took approximately 265 s per epoch. An early stopping condition regulated the number of epochs, and training stopped after the validation loss defined by categorical cross-entropy hadn’t improved for ten epochs. Adam with default settings was used as an optimizer^52^.

#### Prediction

Seamless resin ducts $\mathscr {P}_D$ and tree-ring boundary $\mathscr {P}_R$ probability maps were obtained at the prediction step via applying a trained model to each image in a sliding window setup to overlapping image tiles (see Algorithm 1). The sliding window of size 128 $\times$ 128 pixels equivalent with a patch size used for model training was moved across the consecutive image tiles, which overlapped by a stride of 20 pixels in both: the horizontal and the vertical direction. The stride was selected empirically to ensure the patches composing an input image fit the GPU memory. Finally, the response of the model to overlapping tiles was averaged and outputted as the final resin ducts and tree-ring boundary likelihood maps $\mathscr {P}_D$ and $\mathscr {P}_R$ respectively.

**Figure 5:**
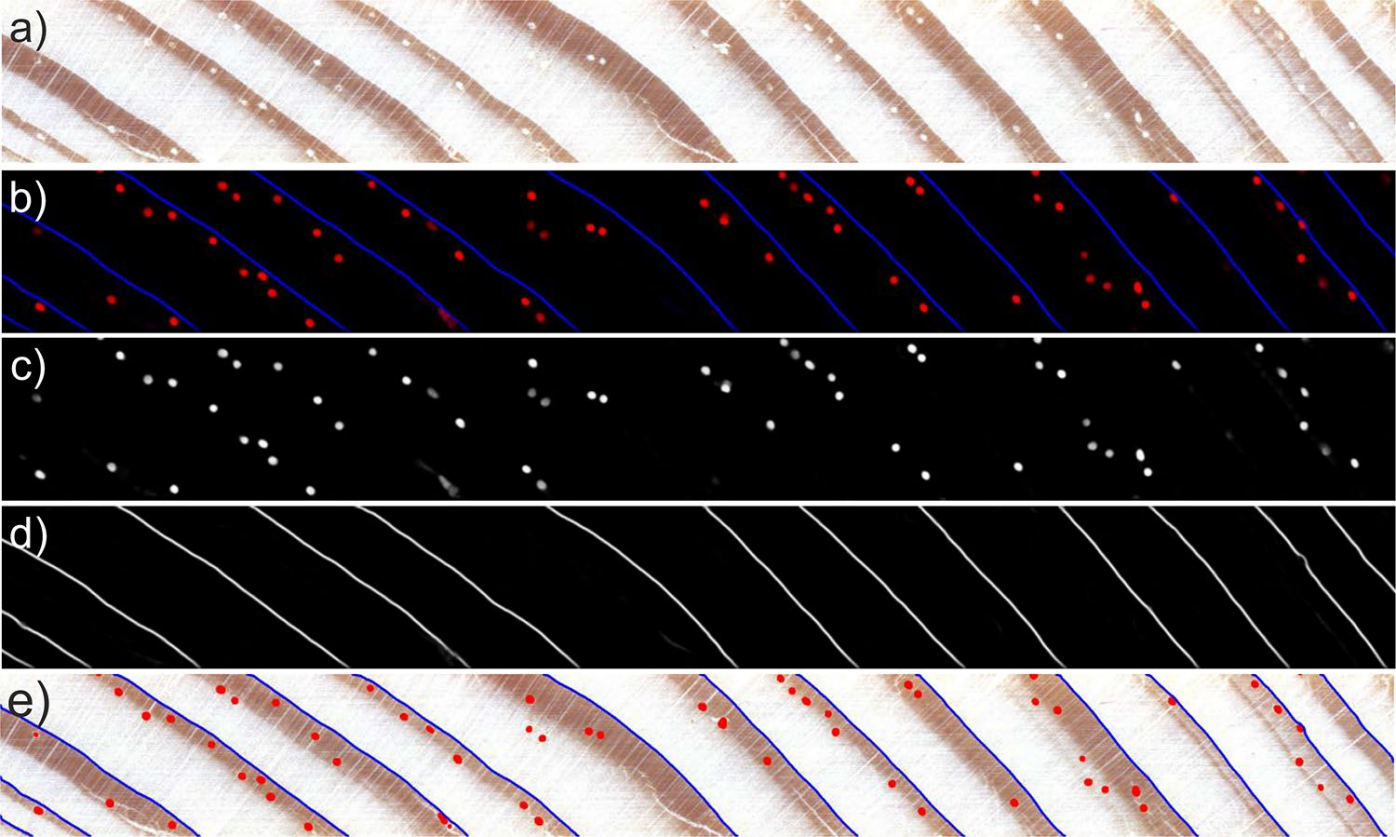
Probability maps generated by the CNN model; (**a**) sample wood core; (**b**) joint probability map of resin canals (red) and tree-ring boundaries (blue); (**c**) resin ducts probability map; (**d**) tree-ring boundary probability map; (**e**) detected resin ducts and tree-ring boundaries overlaid on the original image.

Sample wood core image and the predicted likelihoods of resin ducts and tree-ring boundaries are presented in Fig. 5.

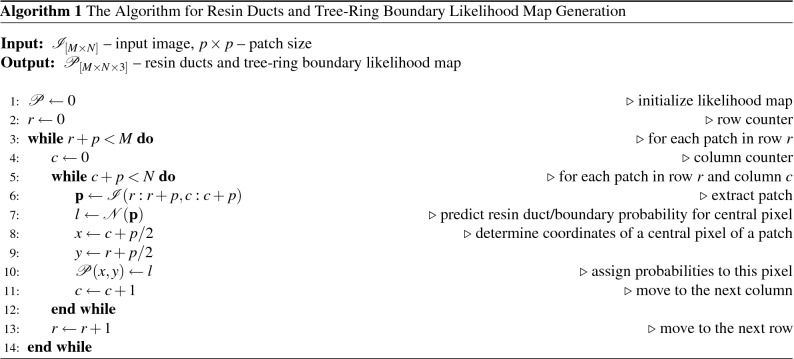



### Resin ducts probability map postprocessing

The resin ducts likelihood map $\mathscr {P}_D$ is utilized to define the exact location and size of the resin ducts $d_i \in \mathscr {D}$. The map is thus binarised $\mathscr {T}: \mathscr {P}_D \rightarrow \{\mathscr {B}$: $\mathscr {B}(x,y) \in \{0, 1\} \wedge \forall _{(x,y): \mathscr {P}(x,y)>t_L} \mathscr {B}_D(x,y) = 1\}$ to obtain regions of the highest resin-duct probabilities. The binarisation is performed adaptively via local thresholding to adapt to weaker (less evident) resin ducts. The local neighborhood of 25 $\times$ 25 pixels of each pixel is considered to determine a threshold $t_L$. This image subregion corresponds roughly to the area of size 0.278 mm$^2$ and was determined through empirical analysis, considering the typical size of a resin duct. In particular, we observed that resin ducts in analyzed images have a diameter of approximately 20–30 pixels. Chosen patch size is, therefore, sufficiently large to differentiate between resin ducts and background while also being small enough to avoid being influenced by larger-scale variations in the image, such as changes in texture. It also allows obtaining the necessary statistics for threshold determination quickly. The threshold equal to the mean probability within the neighborhood increased by a small constant to increase noise resistance is applied to find the local maxima of resin-duct probabilities.

The last step is shape-based filtering of the detected objects to remove regions that are not circular. If an object’s circularity is below 0.9 and its area is smaller than half of the average of all objects, such an object is considered an outlier and removed from the resulting binary map of resin ducts.

**Figure 6:**
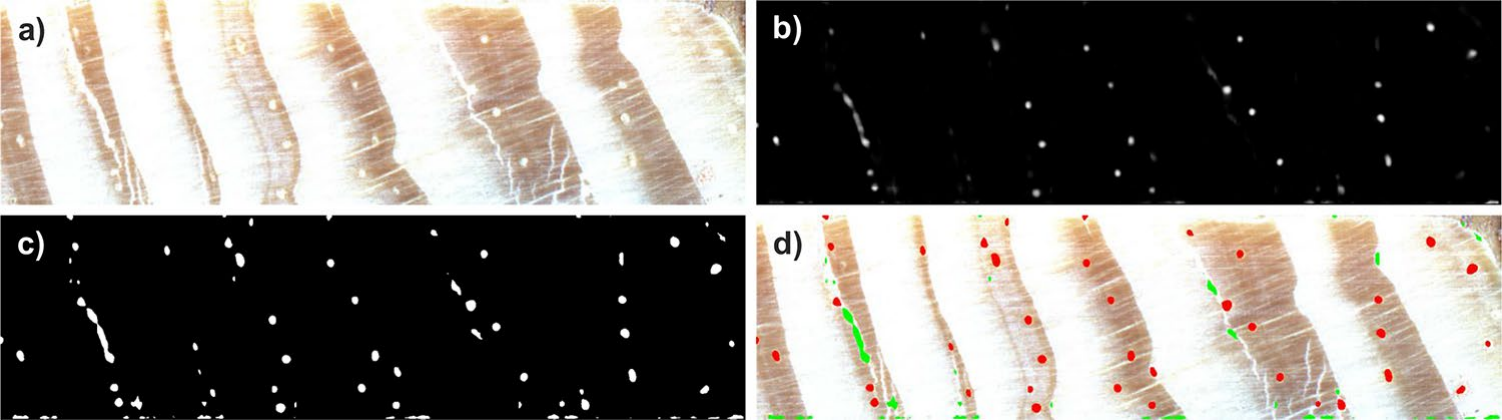
Consecutive steps of resin duct likelihood map postprocessing; (**a**) sample wood core image; (**b**) resin ducts likelihood map; (**c**) binarised resin ducts likelihood map; (**d**) detected resin ducts (in red) overlaid on a wood sample image together with regions removed by shape-based filtering (in green).

Consecutive steps of the shape-based filtering are presented in Fig. 6, including sample wood core section (Fig. 6a), resin ducts likelihood outputted by the CNN model, original (Fig. 6b) and after binarisation (Fig. 6c), and finally detected resin ducts shown in red and outliers shown in green (Fig. 6d).

### Tree-rings segmentation

**Figure 7:**
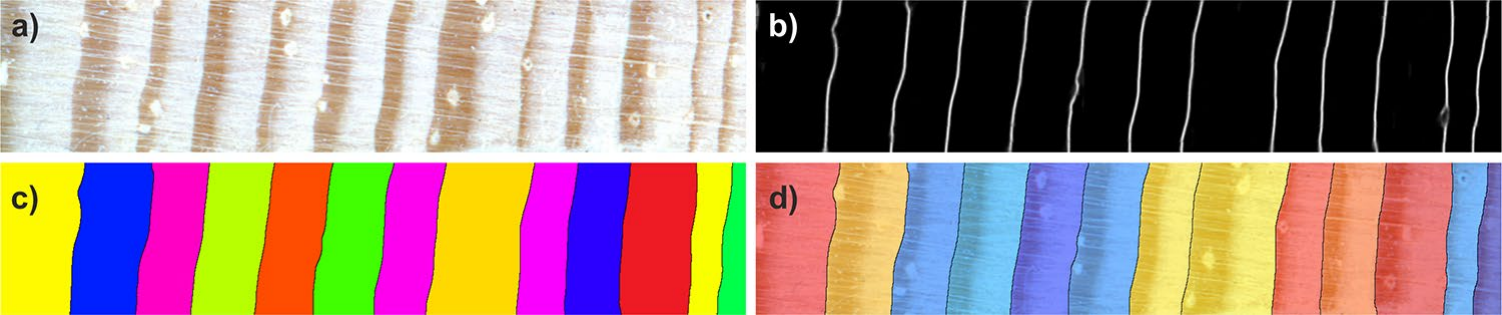
Consecutive steps of tree-ring boundary likelihood postprocessing; (**a**) sample wood core image; (**b**) tree-ring boundary likelihood map; (**c**) tree rings after segmentatation (each shown in different color); (**d**) tree rings overlaid on an input wood sample image.

For tree ring segmentation, tree-ring boundary likelihood maps $\mathscr {P}_B$ are utilized (see Fig. 7b). First, similar to the previous step, local adaptive thresholding $\mathscr {T}: \mathscr {P}_B \rightarrow \{\mathscr {P}_B': \mathscr {P}_B'(x,y) \in (0, 1) \wedge \forall _{(x,y): \mathscr {P}_B'(x,y)<t_L} \mathscr {P}_B'(x,y) = 0\} \}$ is applied to define the boundary candidates, i.e., regions with the highest probability corresponding to the tree ring boundaries (see Fig. 7c). Again, mean intensity within the pixel neighborhood of size 25 $\times$ 25 pixels is considered a threshold $t_L$. The window size was determined experimentally to balance the trade-off between accurately distinguishing tree rings from the background and avoiding over- or under-segmentation, particularly in the case of narrow, densely located tree-ring boundaries. However, the map is not binarised, but the pixels of intensity smaller than the threshold are set to 0. The remaining pixels are left unchanged. Such a map is next subjected to watershed segmentation. It results in regions that roughly correspond to the rings. The preprocessing step diminishes the level of over-segmentation. However, the over-segmentation can still be observed for some rings. Therefore in the last step, region merging is performed. All regions smaller than a predefined threshold (here set to 1500 pixels selected experimentally) are joined with the larger neighboring regions. If the region is neighboring to several regions, it is included in the region with the longest common boundary (cf. Algorithm 2).

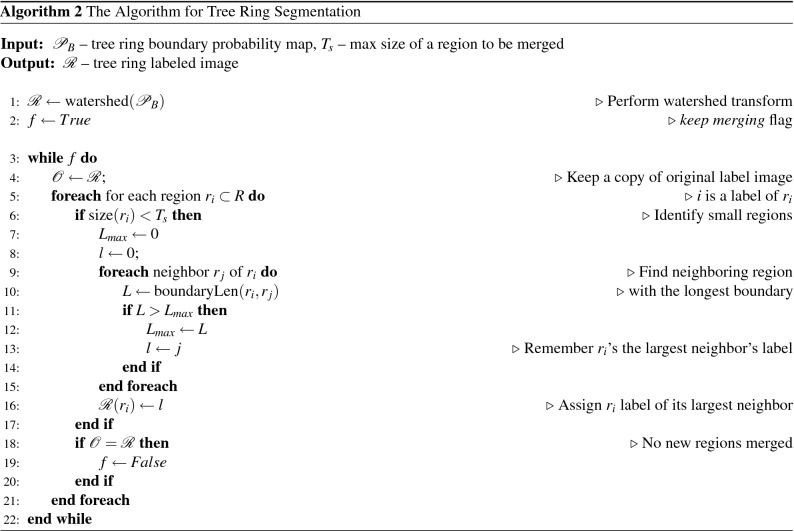



Finally, watershed transform is applied to the binary image of linked tree-ring boundaries to extract connected components $r_i \in \mathscr {R}$ label them with unique labels $i\in [0, K]$ where *K* is the number of the detected tree rings (see Fig. 7 ).

### Measurement

The segmented maps of the tree rings $\mathscr {R}$ and resin ducts $\mathscr {B}$ are next utilized for the linear and surface measurements of these structures.

#### Ring width

**Figure 8:**
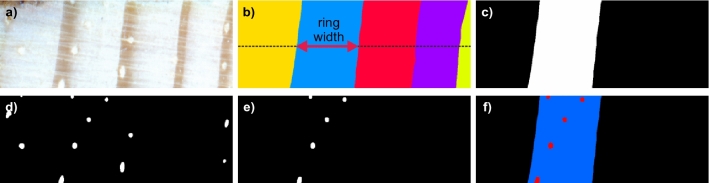
Consecutive steps of tree rings and ducts properties measurement; (**a**) sample wood core segment; (**b**) corresponding tree rings map with the width of a third ring marked; dashed line presents core horizontal centerline; (**c**) a binary mask of the third ring; (**d**) binary mask of ducts found in the wood core segment; (**e**) ducts located within the third ring (logical AND of (**c**) and (**d**)); (**f**) the third ring with ducts contained. The measurement procedure is repeated for each detected found in the wood core.

The ring widths in wood core images (see Fig. 8a) are measured along the horizontal line traversing the core center (see Fig. 8b). The ring boundary curvature is not accounted for by using the core center, but the purpose here is comparison between the automated and expert placed ring boundaries not chronology development. Since each tree ring region $r_i \in \mathscr {R}$ was assigned a unique label $i \in [1,K]$ where *K* is the number of rings, the procedure counts the number of consecutive connected pixels located along the core centerline and assigned one label. Since the core images were scanned at the resolution of 2400 dpi, the resulting number is then multiplied by 25.4/2400 to obtain the core width in millimeters.

#### Ring area

A ring is connected component $r_i$ with all pixels assigned a label *i*. The ring area is thus determined by counting the number of pixels assigned to one label (see Fig. 8b,c) and multiplying it by $(25.4/2400)^2$ to obtain the core area in square millimeters.

#### The number of ducts per tree ring

To determine the number of ducts contained in the ring $r_i$ is first masked, with a mask $m_i = \{(x,y): \mathscr {R}(x,y) = i\}$ where $i \in [1, K]$ and *K* is the number of rings (see Fig. 8c). Then the logical AND operation is computed between the resulting ring mask $m_i$ and the binary ducts map $\mathscr {R}(x,y)$ (see Fig. 8d). The number of connected components in the resulting logical image $d_i = m_i \wedge \mathscr {R}_R(x,y)$ constitutes the number of ducts in the ring $r_i$ (see Fig. 8e,f).

#### Tree ring coverage by ducts

The fraction of the ring covered by ducts is determined by dividing the total area of ducts in image $d_i$ by the total area of ring $r_i$. The areas of both ducts and tree rings can be determined by counting non-zero pixels in images $r_i$ and $m_i$, respectively.

## Results

### Visual results

**Figure 9:**
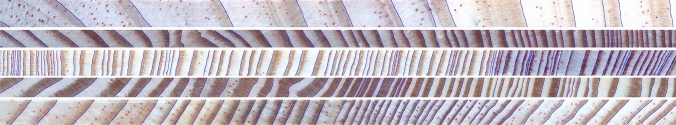
Visual results of resin ducts and tree-ring boundary detection. The species represented by the samples are as follows (from top to bottom): *Pinus palustris* Mill. (PIPA2 or PIPA), *Pinus pungens* Lamb. (PIPU5 or PIPU), *Pinus rigida* Mill. (PIRI), *Pinus taeda* L. (PITA), *Pinus virginiana* Mill. (PIVI2 or PIVI).

Figure 9 presents visual results of resin ducts and tree-ring boundary detection in sample wood core segments using the proposed pipeline. The detected tree-ring boundaries are marked blue, while the detected resin ducts are outlined in red. Images representing each of the five considered species were selected for presentation.

### Numerical results

The numerical assessment of resin duct detection and tree-ring boundary detection was performed by comparing the output of the proposed pipeline with the expert annotations of both considered structures. Both for resin ducts and tree-ring boundaries, the procedure included counting: (i) true positives (TP), i.e., structures correctly detected by the proposed pipeline, (ii) false negatives (FN), i.e., structures marked by an expert but missed by the proposed approach, and (iii) false positives (FP), i.e., false resin ducts/tree-ring boundaries introduced by the automatic approach.

Additionally, sensitivity $SEN =\frac{TP}{TP+FN}$ and precision $PREC = \frac{TP}{TP+FP}$ were determined. Specifically, sensitivity describes how good a proposed approach is at detecting considered structures correctly, while precision provides a ratio of the positively classified structures being relevant.

#### Resin ducts detection

The numerical assessment of the proposed approach in the resin ducts detection task is presented in Tables 2 and 3. Particularly, Table 2 presents global scores obtained for each of the three testing folds, while Table 3 additionally provides scores averaged over each of the considered wood species. A resin duct was considered true positive if it overlapped at least partially with the corresponding expert annotation.

**Table 2:** The resin duct detection performance—global scores for each testing fold.

	TP (correct)	FN (missed)	FP (false)	SEN	PREC
Fold 1	7897	1225	2384	0.87	0.77
Fold 2	6827	1384	2329	0.83	0.75
Fold 3	6290	1112	2022	0.85	0.76
Global	21014	3721	6735	0.85	0.76

**Table 3:** The resin duct detection performance—scores averaged per species for each testing fold.

Species	SEN				PREC			
	Fold 1	Fold 2	Fold 3	Average	Fold 1	Fold 2	Fold 3	Average
PIPA	0.88	0.73	0.86	0.83	0.69	0.64	0.70	0.68
PIPU	0.85	0.84	0.85	0.85	0.74	0.79	0.79	0.77
PIRI	0.87	0.84	0.82	0.85	0.79	0.76	0.77	0.77
PITA	0.96	0.92	0.90	0.93	0.80	0.72	0.84	0.79
PIVI	0.77	0.73	0.85	0.78	0.72	0.72	0.65	0.69

#### Tree-ring boundary detection

The numerical assessment of the proposed approach in the tree-ring boundary detection task is presented in Tables 4 and 5. Similarly to resin ducts, global scores (cf. Table 4) and scores averaged per each considered species (cf. Table 5) are presented. The tree-ring boundary was considered detected if it overlapped at least partially with the corresponding tree-ring boundary annotated by an expert.

**Table 4:** The tree-ring boundary detection performance—global scores for each testing fold.

	TP (correct)	FN (missed)	FP (false)	SEN	PREC
Fold 1	2680	159	28	0.94	0.99
Fold 2	2484	275	11	0.90	1.00
Fold 3	2289	173	36	0.93	0.98
Global	7453	607	75	0.92	0.99

**Table 5:** The tree-rings detection performance—scores averaged per species for each testing fold.

Species	SEN				PREC			
	Fold 1	Fold 2	Fold 3	Average	Fold 1	Fold 2	Fold 3	Average
PIPA	0.83	0.69	0.95	0.82	0.88	0.97	0.84	0.89
PIPU	0.97	0.96	0.98	0.97	1.00	1.00	0.99	1.00
PIRI	0.93	0.90	0.90	0.91	1.00	1.00	1.00	1.00
PITA	0.95	0.92	0.95	0.94	0.96	0.99	0.95	0.97
PIVI	0.96	0.87	0.94	0.92	0.99	1.00	1.00	1.00

#### Measurement

**Figure 10:**
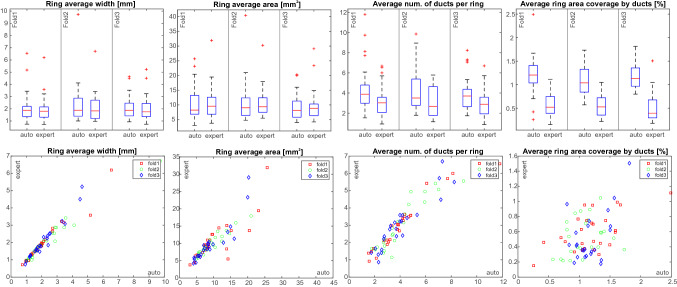
The results of tree rings and duct properties automatic measurement (series *auto*) compared with the values obtained from expert annotations (series *expert*). The measurement type is given in the plot title. Box plots represent the distribution of average measures obtained for cores in each testing fold.

The results of tree rings and duct properties measurements are summarised in Fig. 10 and Tables 6 and 7. Particularly, Fig. 10 presents box plots of the distributions of the tree ring average width, the tree ring average area, the average number of ducts per tree ring, and an average tree ring area coverage by ducts obtained for cores in each testing fold using the proposed pipeline (series *auto*). The results are compared with the corresponding measurements derived from expert annotations (series *expert*). The bottom panel also compares individual measurements obtained for each core using both considered measurement methods. The results are supplemented with a squared correlation coefficient between the measures obtained from a fully automatic pipeline and the measures derived from expert annotations shown in Table 6. Finally, the mean average values of the considered rings and ducts parameters are presented in Table 7.

**Table 6:** $R^2$ correlation coefficient between fully automatic measurement and measurements derived from expert annotations.

Parameter name	Fold1	Fold2	Fold3	Global
Tree ring average width	0.93	0.84	0.95	0.91
Tree ring average area	0.67	0.88	0.72	0.79
Avg. num. of ducts per tree ring	0.60	0.48	0.52	0.54
Avg. tree ring coverage by ducts	− 1.70	− 3.90	− 7.25	− 3.14

**Table 7:** Comparison of the mean average values of the determined rings and ducts parameters.

Parameter name	Fold 1		Fold 2		Fold 3		Global	
	Auto	Expert	Auto	Expert	Auto	Expert	Auto	Expert
Tree ring avg. width (mm)	2.11	1.98	2.44	2.12	2.14	2.06	2.22	2.05
Tree ring avg. area (mm$^2$)	10.11	10.54	10.86	10.78	9.19	9.98	10.06	10.44
Avg. num. of ducts per tree ring	3.36	3.19	4.31	3.13	4.02	3.01	4.24	3.11
Avg. tree ring coverage by ducts (%)	1.12	0.59	1.07	0.53	1.18	0.52	1.15	0.55

#### Processing time

Finally, the time required for automatic ducts and tree-ring boundaries detection using the proposed pipeline was compared to the time spent by an expert annotating these structures in wood core images. The recorded times are shown in Table 8. For each considered species, a total and average time in seconds spent on processing the cores (manually or automatically) is given.

**Table 8:** Time in seconds spent processing the cores representing each of the considered species. Series *Manual* refers to time spent by an expert annotating the cores. Series *Auto* refers to the time required by the proposed pipeline.

Species	Num. images	Auto		Manual	
		Total time (s)	Avg. per core (s)	Total time (s)	Avg. per core (s)
PIVI	18	83	4.61	18990	1055.00
PITA	13	96	7.38	28482	2190.92
PIRI	30	169	5.63	32057	1068.57
PIPU	10	53	5.30	8071	807.10
PIPA	3	22	7.33	2496	832.00
Total	74	423	5.72	90096	1217.51

## Discussion

The results of the proposed pipeline are auspicious. The models’ performance in resin duct detection is high. The average sensitivity at the level of 0.85 indicates that the convolutional neural network detected about 85% of the resin ducts marked by an expert. On the other hand, precision at the level of 0.76 means that the model introduced a noticeable number of false ducts. However, a closer inspection of these ducts revealed that some could be deemed missed by an expert but found by the model. As a result, the CNN model seems to perform somewhat better than the error would indicate when detecting the resin ducts. A quality control step would be beneficial for improving the reliability of the train data and thus improving the model’s performance in the resin duct detection task.

Differences in the model’s performance between species can be observed. The most challenging was PIVI. For this species, the model misdetected, on average, 22% of the ducts simultaneously introducing the highest number of false ducts. Narrow rings with overall small resin duct size in this species could have made detection more difficult. On the other hand, the PITA seems to be the easiest for automatic processing. For this species, the CNN model scored the highest sensitivity (0.93) and specificity in the resin ducts detection task. PITA has high growth rates relative to the other species resulting in wide rings with larger and clear resin duct boundaries that might have simplified the detection task.

**Figure 11:**
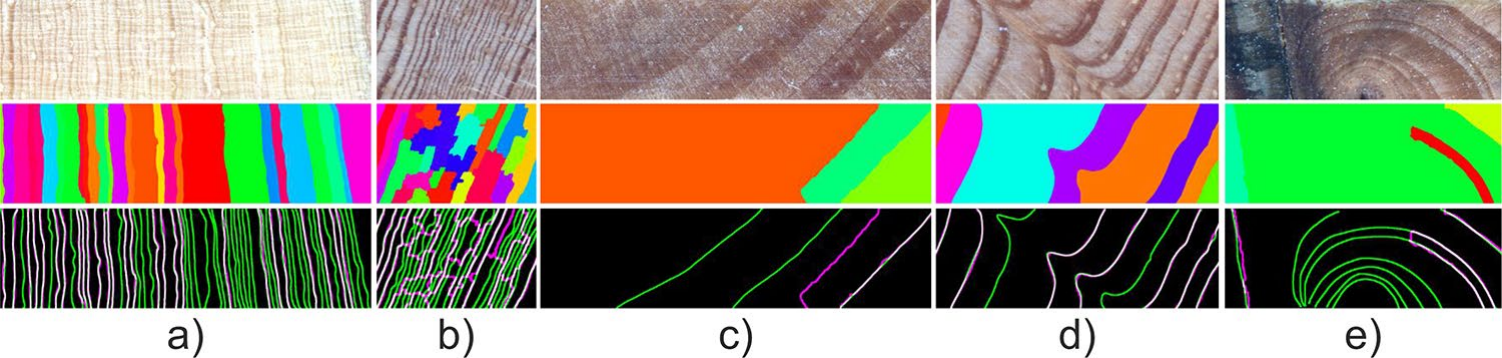
Tree-rings boundary detection—common errors; top panel—patches of original images; middle panel—results of rings segmentation; bottom panel—expert versus automatic result (green—missed boundaries, magenta—false boundaries, white—expert and automatic boundaries overlapping.

The CNN model was even more accurate when detecting the tree-ring boundaries. In total, it missed only 8% of these structures, with a very low rate of false tree-ring boundaries (precision on average equal to 0.99). The CNN model performed very well when detecting linear and regular tree-ring boundaries. Such boundaries constituted the training set predominantly. On the other hand, most missed boundaries were curly, curved, and arch-shaped (cf. Fig. 11c–e). Such boundaries were underrepresented in the training set, which resulted in model’s worse performance in relation to them. The CNN model also mostly failed when detecting boundaries of densely packed and thin tree rings. This, in turn, resulted in the over-segmenting of these tree rings (cf. Fig. 11a,b). Again, the performance of the model when used for tree-ring boundary detection varied depending on the species. The highest scores (SEN of 0.97 and PREC of 1.0) were obtained for PIPU. In comparison to the other pines in the dataset, PIPU had clear ring boundaries with good contrast and very few ”false” rings. On the other hand, the most challenging was PIPA since, for this species, the model performed worst, with 18% of missed tree-ring boundaries and the highest number of false boundaries. PIPA in comparison to the other pines in the dataset had low contrast yielding less distinct ring boundaries.

Good performance of tree-ring boundary detection translates directly into accurate measurements of tree-ring widths. As shown in Fig. 10 automatic width measurements agree with the measurements derived from expert annotations. This is confirmed by the $R^2$ coefficient on average equal to 0.91, for fold 3 reaching even the value of 0.95 (cf. Table 6). Also, the mean absolute error of the mean average tree ring width does not exceed 10% (cf. Table 7) which can be considered a very good result.

More challenging than the tree-ring boundary detection is the tree rings segmentation aimed at extracting connected components bounded by two tree-ring boundaries and corresponding to consecutive rings. The accuracy of tree ring segmentation translates into tree-ring area measurement accuracy. One challenge to this problem is that the detected tree-ring boundaries are often discontinuous, weak, or do not span the core width. As a result, some neighboring tree rings may be merged, increasing the average core area. On the other hand, some closely located, dense tree-ring boundaries may connect in the middle of the core width, resulting in over-segmentation (cf. Fig. 11b). Still, the results of core area measurement can be considered accurate. The $R^2$ correlation coefficient between the ring area determined fully automatically and derived from expert annotations is high at the level of 0.79 on average, for fold 2, reaching even the value of 0.88 (cf. Table 6). The ring over-segmentation and under-segmentation errors seem to compensate for each other since the global difference between the mean average area determined automatically and based on expert annotations does not exceed 4% (cf. Tab 7).

The remaining two statistics, i.e., the average number of ducts per ring and ring area coverage by ducts, are visibly less accurate. The reason is that they cumulate errors in both duct detection and tree ring segmentation. First, the resin duct detection mechanism overestimates the total number of ducts (the number of false negatives is less than the number of false positives). Next, the tree ring segmentation algorithm tends to over-segment the thin and densely located tree rings. Consequently, the average number of ducts per ring is overestimated by c.a. 36% compared to stats derived from expert manual annotations. It is equivalent to about one additional (false) resin duct in a statistical tree ring (cf. Table 7). Still, the correlation between automatic and expert measurements is quite high, with the $R^2$ correlation coefficient at the average level reaching 0.55 (cf. Table 6).

**Figure 12:**
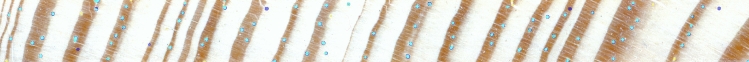
Resin duct detection errors; cyan—regions where automatic and expert annotations match, navy—regions detected by an automatic pipeline but not marked by an expert; yellow—regions marked by an expert but missed by an automatic pipeline.

The highest error corresponds to the measurement of tree ring area average coverage by ducts. Apart from the factors mentioned above, the proposed pipeline seems to overestimate the ring size compared to expert annotations. This effect is presented in Fig. 12, where ducts detected by the proposed pipeline (shown in navy) are visibly larger than the ducts annotated by an expert (the overlapping regions are shown in cyan). The issue could be fixed by the dedicated postprocessing of the ring detection results or by developing the dedicated ducts segmentation algorithm.

As with every machine learning-based tool, our method poses some challenges and limitations^53^. The proposed pipeline requires a substantial number of annotated resin ducts and tree rings to train the model. This study does not test how many more resin ducts or tree rings would improve the error rate. The accuracy of the proposed method may be affected by the quality and resolution of the wood images, the quality of expert annotations, and the variability of resin duct structures and their relationships with tree rings across different Pinus species. The proposed method may require further validation to assess its performance and reliability on a wide range of wood samples including sections from dead trees. Future research opportunities include testing a larger dataset with species specific models or attempting to detect and measure other features of tree rings (e.g., fire scars or frost rings).

Finally, the proposed pipeline significantly reduces the core processing time (cf. Table 8). The time spent by an expert on annotating resin ducts and tree-ring boundaries in a wood core image ranged from 120 to 5465 s, with an average of 1217 s (c.a. 20 min) per core. The total time spent by an expert on annotating all 74 cores was about 25 h. The time required by the proposed pipeline to perform this task automatically was 423 s (c.a. 7 min), with an average of fewer than 6 s per core. The automatic processing was then more than 200 times faster than an expert. It is a significant improvement, even if some manual corrections are still required to fix the results of automatic resin ducts and tree-ring boundary detection. The proposed method can significantly reduce the time and effort required for resin duct and tree-ring analysis, making it more accessible to researchers and practitioners in dendrochronology.

## Conclusions

Here we have presented new automatic methods that can be further developed to aid future research efforts into resin ducts. The convolutional neural network, and in particular the modified AttUNet model implemented in a patch-based set up, provides a useful method of automated detection of both tree rings and resin ducts in wood core images. Postprocessing of the detected structures allows determination of resin duct statistics in relation to the tree-rings with a relatively high accuracy.

The proposed pipeline offers several advantages over traditional manual methods. Firstly, it is much faster, as it does not require a human operator to mark and measure the resin ducts. This results in a significant reduction in processing time, especially for large datasets. Secondly, the pipeline eliminates the possibility of human error in marking and measuring the resin ducts, resulting in more accurate and consistent detection. This is particularly important when dealing with large datasets, where the manual method may be prone to variation and errors, depending on the operator’s experience and judgment. Future research could include an investigation of which false-positive resin duct detection by the model were actually true ducts overlooked by an expert. The task of marking resin ducts and tree rings requires sustained concentration, therefore some degree of human error would be expected that is not accounted for in the model performance scores. Finally, the pipeline allows for standardized analysis of resin duct characteristics regarding the tree ring area to which they belong. This is an improvement over traditional methods, which do not account for the location and distribution of the resin ducts within the tree rings. Standardization makes comparing results across different samples and studies easier, leading to more robust and reliable conclusions. The increased volume of data facilitated by the method can provide valuable insights into the environmental conditions and growth patterns of Pinus trees, which can be used to study and monitor the effects of climate change and other factors on forest ecosystems.

Resin duct characteristics and oleoresin production have been shown to be important defense mechanisms for certain tree species in the genus *Pinus*^54^. Measuring resin duct characteristics of trees can provide trends related to the effect of management actions at specific points in time along with responses to pests and pathogens. Despite recent progress in measuring and quantifying resin ducts^4,55^, research on this important defense mechanism has been hampered by the difficult and time consuming task of manually delineating duct areas and tree ring boundaries. The automation presented here represents a large improvement in the speed of measuring these attributes for pine tree wood cores. Methods outlined in this study could be incorporated into future dendrochronological software applications to improve workflow. The method can be adapted and optimized for other species and types of wood, potentially enabling new applications in fields such as wood science, ecology, and forestry.

## Data Availability

The datasets used and/or analysed during the current study available from the corresponding author on reasonable request.
